# AlphaTracker: a multi-animal tracking and behavioral analysis tool

**DOI:** 10.3389/fnbeh.2023.1111908

**Published:** 2023-05-30

**Authors:** Zexin Chen, Ruihan Zhang, Hao-Shu Fang, Yu E. Zhang, Aneesh Bal, Haowen Zhou, Rachel R. Rock, Nancy Padilla-Coreano, Laurel R. Keyes, Haoyi Zhu, Yong-Lu Li, Takaki Komiyama, Kay M. Tye, Cewu Lu

**Affiliations:** ^1^Department of Computer Science, Shanghai Jiao Tong University, Shanghai, China; ^2^Zhiyuan College, Shanghai Jiao Tong University, Shanghai, China; ^3^Media Arts and Sciences, Massachusetts Institute of Technology, Cambridge, MA, United States; ^4^Department of Neurobiology, Center for Neural Circuits and Behavior, University of California, San Diego, La Jolla, CA, United States; ^5^Department of Neurosciences, University of California, San Diego, La Jolla, CA, United States; ^6^Department of Psychological and Brain Sciences, Johns Hopkins University, Baltimore, MD, United States; ^7^Salk Institute for Biological Studies, La Jolla, CA, United States; ^8^Department of Neuroscience, University of Florida, Gainesville, FL, United States; ^9^Howard Hughes Medical Institute, The Salk Institute, La Jolla, CA, United States; ^10^Shanghai Artificial Intelligence Laboratory, Shanghai, China

**Keywords:** neuroscience, computer vision, animal behavior, animal tracking, behavioral clustering

## Abstract

Computer vision has emerged as a powerful tool to elevate behavioral research. This protocol describes a computer vision machine learning pipeline called AlphaTracker, which has minimal hardware requirements and produces reliable tracking of multiple unmarked animals, as well as behavioral clustering. AlphaTracker pairs a top-down pose-estimation software combined with unsupervised clustering to facilitate behavioral motif discovery that will accelerate behavioral research. All steps of the protocol are provided as open-source software with graphic user interfaces or implementable with command-line prompts. Users with a graphical processing unit (GPU) can model and analyze animal behaviors of interest in less than a day. AlphaTracker greatly facilitates the analysis of the mechanism of individual/social behavior and group dynamics.

## 1. Introduction

### 1.1. Development of the protocol

The study of animal behavior can be dated back to the nineteenth century when most researchers focused on observing natural behaviors (Darwin, 1872; Tinbergen, 1963). While reductionist behavioral paradigms are still widely used to study specific aspects of behavior in a controlled manner, allowing animals to freely explore spaces and to exhibit complex behaviors greatly expands our understanding of system neuroscience (Kabra et al., 2013; Wiltschko et al., 2015; Mathis et al., 2018; Pereira et al., 2020a; Padilla-Coreano et al., 2022). Ethological behavioral research challenges our ability to quantify behavior and draw statistically meaningful conclusions with traditional tracking methods and manual annotations (Berman, 2018). Social behavior is even more challenging due to the difficulty for a human to observe multiple animals simultaneously. Traditional animal tracking software suffers from noisy prediction of animal poses and confusion between multiple, seemingly identical animals. In addition, there remains a large gap between tracking animal keypoints and the quantification and understanding of observed behaviors.

Here we present a pipeline that allows reliable tracking of multiple near-identical mice and the subsequent behavioral analysis *via* unsupervised methods. AlphaTracker enables multi-animal tracking of videos recorded *via* a webcam, rendering this tool convenient and affordable for the laboratory setting. To facilitate accessibility to AlphaTracker for individuals without access to a GPU, we also provide a Google Colab version. We also provide an unsupervised behavioral clustering algorithm for the unbiased identification of behavioral motifs, the results of which can be further inspected with customized functions in a Jupyter notebook and a web-based user interface.

### 1.2. Applications of the method

AlphaTracker demonstrates excellent accuracy in diverse backgrounds and setups. Our test cases include both wild-type C57BL/6 black mice and mice with optical fibers and *in vivo* recording head stages, the keypoints of which are often occluded in keypoint tracking for existing software. AlphaTracker demonstrates robust performance with various backgrounds including home cages, metal operant conditioning chambers, and open fields. Our tracking algorithm shows better accuracy and precision than that of two different humans annotating the same dataset. It supports not only top-view cameras, but also cameras installed at an angle, and low-resolution webcams (e.g., 675 p), making simultaneous monitoring of many animals financially tractable.

AlphaTracker shows robust performance in tracking multiple animals and identifying social behavior. Traditional methods of attaching markers or changing fur colors can affect the natural behavior of animals and thus confound the research. Our toolbox was developed specifically for markerless tracking in multi-animal paradigms. Such simultaneous observation of multiple freely-behaving animals makes it a handy tool for social behavioral neuroscience research.

Our unsupervised behavioral clustering bridges the gap between current keypoint-tracking techniques and the challenge of behavior comprehension. Clusters identified by our behavioral clustering algorithm correspond greatly to human assignment (Adjusted Rand Index of 0.201186, random assignment has the ARI of 0.003451). We identified seven individual behaviors: walking, digging, sniffing, rearing, turning, face grooming, and body grooming, and nine social behaviors including following, chasing, anogenital sniffing, face sniffing, and social rearing. We envision our clustering algorithm, with proper training, demonstrating extended application in tracking other animals such as marmosets, fish, and humans with proper training.

## 2. Materials and methods

### 2.1. Materials

#### 2.1.1. Software

- Operating system: Linux (Ubuntu 16.04 LTS, 18.04 LTS), or Windows (10) (Windows only supports applying the tracking model, but not training the neural network-based model).
- Anaconda: a free and open-source distribution of the Python programming language. AlphaTracker is written in Python 3.8 and is not compatible with Python 2.
- AlphaTracker: an actively maintained toolbox freely available at: https://github.com/MVIG-SJTU/AlphaTracker. Instructions in this paper are based on this version. Recently, we provided a sister version of our package on GitHub (https://github.com/Tyelab/AlphaTracker2) which is more friendly to Windows users as it provides a Python wrapper for the DarkNet in the YOLO package, the original version of which is a C-based toolbox that must be compiled on Linux systems. With the goal of offering real-time tracking, this version also adds some flexibility in processing speeds by offering options for lighter-weight networks like Mobile Net in place of the ResNet backbone with the goal of offering real-time tracking.
- PyTorch: an open-source software library for Deep Learning. Our toolbox has been tested on PyTorch 1.8.0.
- Nvidia Driver: a driver software with a version higher than 450 is required to run our model on a computer with Nvidia GPU card, available at: https://www.nvidia.com/download/index.aspx.
- Jupyter Notebook: a web-based interactive computing platform available at: https://jupyter.org/install.
- Data annotation toolbox Sloth: an open-source software for labeling keypoints and identities of objects, provided as part of our toolbox.

#### 2.1.2. Hardware

- Computer: Windows and Linux all can be used for labeling data, performing behavioral clustering, and evaluating trained tracking models. For training the tracking model, desktops/cloud servers with GPU access are required. We recommend >= 32 GB of RAM on the system for CPU analysis.
- GPU: GPU is required for training the tracking model. We recommend having a GPU with >= 8 GB memory, such as the NVIDIA GeForce 1,080 or 2,080. Alternatively, our toolbox can also be used on cloud computing services with GPU support (e.g., Google Cloud/Amazon Web Services).
- Camera: Our toolbox supports both color and grayscale videos, and even infrared light. Though we demonstrate decent performance on images with low resolution, we recommend cameras with a resolution of >=1,080 p for the best performance. We used Logitech C930e cameras for data acquisition in this paper.

#### 2.1.3. Equipment setup

Users can either install our toolbox locally or on Google Colab. We recommend users with GPU access run AlphaTracker locally (local desktop or lab server) to avoid reinstallation of dependency upon restarting Colab.

For GPU support, NVIDIA drivers should be installed (see the previous subsection for details). Windows users double-click the downloaded .exe file to install it. Linux users first navigate to the folder with the “.run” file after downloading, open the terminal and type the following command in the terminal:

1. cd path/of/driver
2. chmod +x ./Nvidia-driver-name.run
3. sudo ./Nvidia-driver-name.run

We recommend both Linux and Windows users install Anaconda for managing packages and environments because Anaconda supports multiple environments with different versions of Python and supporting libraries. This avoids version mismatch with existing packages and libraries of the operating system. Follow the instructions at https://docs.anaconda.com/anaconda/install/ to install Anaconda.

Windows users should install Git Bash by downloading from https://git-scm.com/downloads. Open the “.exe” file named “Git Bash” and run all commands within Git Bash.

### 2.2. Algorithm

**Figure 1:**
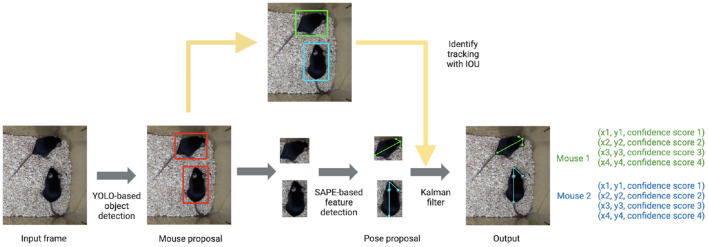
AlphaTracker architecture and pipeline. The AlphaTracker architecture consists of three main components: object detection using YOLO, pose estimation using Single Animal Pose Estimation (SAPE), and identity tracking based on intersection over union (IOU) with error correction *via* Kalman filter. It outputs bounding boxes and user-defined keypoints for each detected animal, along with confidence scores for these predictions.

The tracking component of the pipeline (AlphaTracker) is adapted from AlphaPose (Fang et al., 2017, 2022), a human pose estimation and tracking library that provides superior performance in both accuracy and efficiency. The algorithm consists of three steps: animal detection, keypoint estimation, and identity (ID) tracking across frames (Figure 1).

First, the algorithm detects the positions of animals in each frame with YOLOv3 (Redmon and Farhadi, 2018) which is a state-of-the-art convolutional neural network designed to detect objects at a high inference speed.

Next, individual animals are cropped out with the bounding box output from YOLOv3. The cropped individual images are fed into Squeeze-and-Excitation Networks (SENet) (Hu et al., 2017) which estimates keypoint positions. For our mouse dataset, we chose the snout, tail base and two ears as our four keypoints. The outputs from SENet include x and y coordinates as well as a confidence score which indicates the reliability of each identified keypoint.

Finally, the algorithm tracks each animal across frames. This presents a significant challenge for many platforms as animals of the same genetic lines often look alike. Traditional Re-ID methods previously implemented (Chen et al., 2018; Ristani and Tomasi, 2018; Feng et al., 2019) tend to fail since such methods typically rely on differences in the appearance of tracked animals. In our pipeline, we propose a novel target association method that captures hierarchical visual information to keep track of the identities of nearly identical animals across frames. We define a descriptor for the position and orientation of the animal from the set of bounding boxes around the entire animal and individual body parts. The similarity score of pairs of descriptors in adjacent frames is calculated according to formula 1.


(1) $$\begin{array}{l} Sim\left(D_{i}^{t},D_{i}^{t+1}\right)=IOU\left(box_{i}^{t},box_{j}^{t+1}\right)+\frac{1}{n}\overset{n}{\underset{k=1}{\sum }}IOU\left(P_{ik}^{t},P_{jk}^{t+1}\right) \end{array}$$

(2) $$\begin{array}{l} IOU\left(box_{1},box_{2}\right)=\frac{\text{AreaOfOverlap}\left(box_{1},box_{2}\right)}{\text{AreaOfUnion}\left(box_{1},box_{2}\right)} \end{array}$$

In formula 1, $D_{i}^{t}$ is the descriptor of animal *i* at frame *t*, $box_{i}^{t}$ is the bounding box of animal *i* at frame *t* predicted by the convolutional neural network. $P_{ik}^{t}$ is the box that wraps the k-th body point of animal *i* at frame *t*. Intersection Overlap Union (*IOU*) is defined by formula 2. After sorting the descriptor similarities in descending order, the descriptors between two adjacent frames with the highest similarity are matched and assigned with the same tracking ID. Across frames, descriptors for dyads are matched with the second-highest similarity score. This procedure is repeated until no animals are left unmatched.

In some cases, the predictions of bounding boxes and body points may not be accurate due to either tracking errors or occlusion. When the users correct the position of keypoints in one frame, we apply Kalman filtering (Kalman, 1960) to model the motion characterized by velocity and acceleration. We then modify the keypoint position predictions in consecutive frames to ensure consistency across time.

Our behavioral clustering classifies mouse behavior with an unsupervised hierarchical clustering algorithm (Wiltschko et al., 2015; Nilsson et al., 2020) for the following reasons: (1) Animal behavioral taxonomy is intrinsically hierarchical in structure. (2) It allows intuitive re-organization of results once the linkage matrix is computed. In our method, we first extract the features of animal behaviors based on the temporal dynamics of poses captured within a 15-frame time window. The 15-frame time window is chosen here since sub-second actions of animals have mean duration ± s.d. = 425 ± 726 ms (Wiltschko et al., 2020). Such features include biologically distinct features such as body length and displacement. When analyzing social behavior, we set one mouse as the reference and calculate the relative motion of the non-reference mouse. Here, users can assign different weights to each feature to reflect feature importance in behavioral clustering. We next apply an agglomerative hierarchical clustering algorithm (Ward, 1963) to cluster clips based on the similarity between their features. Finally, a customized web-based UI allows easy inspection and modification of clustering results.

### 2.3. Methods

Our protocol consists of several stages: installation, preparing training datasets (Section 2.3.2), training the tracking model (Section 2.3.3), running the tracking model (Section 2.3.4), inspecting the tracking results with UI (Section 2.3.7), behavioral clustering (Section 2.3.6), and inspecting clustering result (section 2.3.7). Users looking for a quick test of our toolbox can skip the training stages (Sections 2.3.2, 2.3.3) and use the pretrained tracking model directly (Section 2.3.4). We also have a tutorial for our Colab version in Section 2.3.8.

#### 2.3.1. Installation

**Figure 2:**
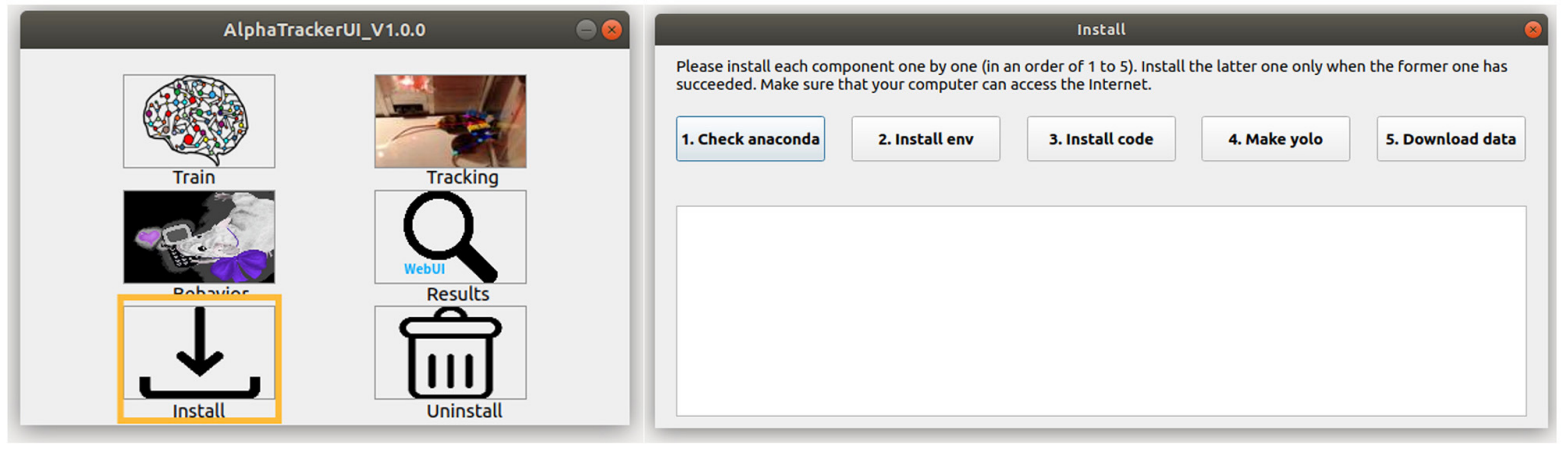
AlphaTracker UI. **(Left)** The AlphaTracker user interface provides six functionalities, including installing/uninstalling, training the tracking model, running the tracking and clustering models, and examining and correcting tracking and clustering results. **(Right)** The installation involves verifying the presence of Anaconda, installing required packages, setting up YOLO, and downloading the model weights and demo data.

1. Download the toolbox *via* the command line. Users can specify a working directory and install it by running the following in the Git Bash terminal.

1. cd path/of/interest
2. git clone https://github.com/MVIG-SJTU/AlphaTracker.git
3. cd AlphaTracker

2. Users can install our toolbox with either command line or *via* a coding-free GUI (Figure 2). Users can run the following commands to install our toolbox *via* the command line. Note that Windows users should first check out the “Windows” branch before the actual installation. Our toolbox creates an Anaconda virtual environment to manage Python dependencies.

1. git checkout windows # Windows users only
2. bash scripts/install.sh

To use our GUI for installation, users need to download a GUI named “main_ui” from https://github.com/MVIG-SJTU/AlphaTracker/releases and save it inside the AlphaTracker folder. Users should: (1) Right-click the GUI app and choose “Properties,” (2) Check the “allow execution” or “allow run as a program” options under the “Permission” tab, (3) Open the main GUI by double-clicking the icon, then (4) Click the “Install” button to run the installation automatically. A video tutorial is available at: https://youtu.be/fQ1bSoAkV5o.

#### 2.3.2. Training dataset preparation

For users hoping to train the model using their own parameters (e.g., animal species, lighting condition), we include an image annotation toolkit to allow customized annotation of training datasets. This toolkit was adapted from an open-source tool Sloth and can be found under the directory ./Tracking/TheAnnotationTool/. This tool has only been tested under Windows. We have also provided a demo training dataset 600 annotated frames of two unmarked mice interacting in a home cage. Users can download this folder from https://drive.google.com/file/d/1TYIXYYIkDDQQ6KRPqforrup_rtS0YetR/view?usp=sharing and proceed to the next section for training.

**Figure 3:**
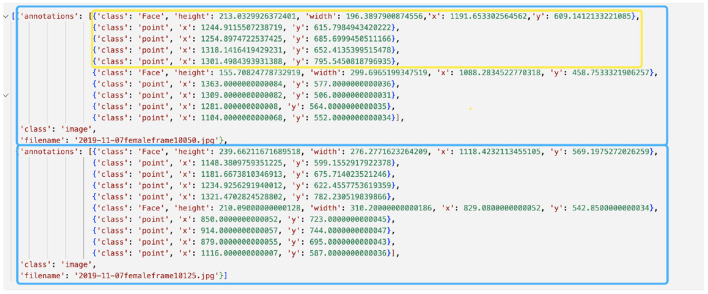
Example annotation JSON file generated by Sloth. The example shows annotations for two frames, represented by blue boxes. Each frame depicts two mice, one of which is highlighted in a yellow box. The yellow-boxed mouse is annotated with a bounding box for the body and four keypoints, which correspond to the head, left ear, right ear, and tail base respectively.

1. Pick representative frames from input videos (>=200 frames are recommended) and save these frames as a folder called “im.” Place the folder under the folder “json.” These frames should be as distinct from each other as possible to cover the posture space. Models that learn from the entire space generalize better during the actual implementation.
2. Click *json/clickme.bat* to create a new json file named *multi_person.json* under the folder *json/*. Move the newly generated JSON file into the directory *json/im/*.
3. Go back to the directory *tool* and click *tool/start.bat*. Select the *multi_person.json* file and click “Open” to load all the images.
4. To meet the input specifications of AlphaTracker, strictly follow the proceeding instructions for image annotation:
  First, choose the “Face” option to generate a red bounding box around the animal of interest on the image. Your definition of a bounding box should be consistent (e.g., if you include the tail in the bounding box, always do so). We recommend only including the tail base for mice because tails are highly flexible and extend to a large area.
  Next, choose the “point” option to label keypoints for that animal. If you have multiple keypoints, it is critical to follow the same annotation order for all the animals (e.g., snout $\overset{}{\underset{}{\to }}$ left ear $\overset{}{\underset{}{\to }}$ right ear $\overset{}{\underset{}{\to }}$ tail base). If you have multiple animals, repeat the process for another animal only after you are completely done with the current animal (i.e., bounding box $\overset{}{\underset{}{\to }}$ all the keypoints) because the order matters.
5. If there is a mistake, you should first select the image on the bottom left of the UI, click the wrongly labeled coordinates, and press “delete.” Make sure to delete all the subsequent coordinates for this frame as well and redo the annotation because the order of annotation is important for our algorithm.
6. Once you are done, press the “save” button to save the JSON file before exiting the program. Rename and move the entire “im/” folder (images and the JSON file) to a safer storage location for later use. As a double-check, the generated JSON file should have the same structure as in Figure 3.

#### 2.3.3. Training the tracking model

1. Users either use our code-free GUI to specify the parameters or modify the settings directly. If using the code-free GUI, click the “Train” button on the main GUI. Select the image folder with the training images and the JSON annotation file in the prompt window. Modify the parameters in the “training” tab. Users can hover their mouse cursor over each parameter for detailed explanations. A video tutorial is available at: https://youtu.be/txjrZiVS4Eo. If modifying the setting directly, specify the parameters in the configuration file ./Tracking/AlphaTracker/setting.py.

Important parameters are as follows:

- *image_root_list*. List of paths to the directories of annotated frames.
- *json_file_list*. List of paths to the corresponding annotation JSON file.
- *num_mouse*. A list of the maximum number of animals that may appear in each of the corresponding image folders.
- *exp_name*. Name for the current project.
- *num_pose*. The number of keypoints for each animal. This must be consistent within the project. If users have videos with different numbers of keypoints per animal, they can set up individual projects to keep the keypoint number consistent within each project.

Depending on the training results, users may need to modify hyperparameters related to training. For example, users can lower the learning rate and increase the number of epochs. However, over-reducing the learning rate may deteriorate tracking quality. Some hyperparameters are explained as follows.

**Figure 4:**
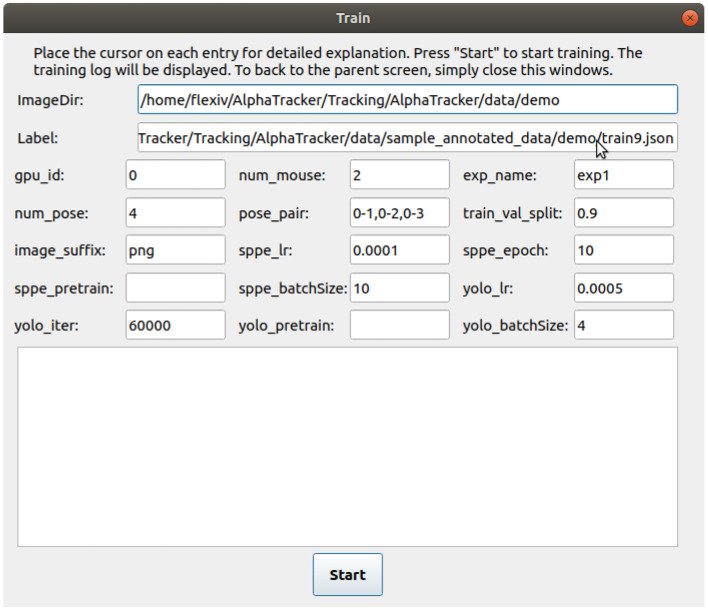
User interface for training the tracking model. The user interface for training the tracking model requires several inputs from the user. The inputs include the path to the labeled images (“ImageDir”), a JSON label file (“Label”), the number of mice in the images (“num_mouse”), the experiment name (“exp_name”), and pose pairs for defining the connections between keypoints to represent the skeleton (“pose_pairs”). Other adjustable parameters such as the learning rate (“sppe_lr,” “yolo_lr”) and batch size (“sppe_batchSize,” “yolo_batchSize”) can be modified as needed.

- *sppe_lr*. Learning rate for the pose estimation module. Default: 1e-4.
- *sppe_epoch*. Training epochs for the pose estimation module. You might need to set a large number when training from scratch.
- *sppe_batchSize*. Batch size for pose estimation. If you encounter an out-of-memory (OOM) error, you may need to reduce the batch size.

2. Train the AlphaTracker model by either clicking the “start” button on the training page after specifying all the parameters (if using the GUI, Figure 4) or by running the following command in the command line.

1. cd path/of/interest/AlphaTracker/
Tracking/AlphaTracker/
2. conda activate alphaTracker
3. python train.py

#### 2.3.4. Running the tracking model

If users have not trained their own models, they can use our pretrained model by setting *exp_name=demo* in the configuration file. This implicitly calls the pretrained model. In case users do not have a video ready to use, we also provide a test video at: ./Tracking/AlphaTracker/data/demo.mp4.

1. Users can set the parameters for tracking by either specifying the parameters in ./Tracking/AlphaTracker/setting.py or using the code-free UI. If using the GUI, click the “Tracking” button on the main GUI and select a video file. Modify the parameters on the “tracking” page. Users can hover the mouse cursor over each parameter for detailed explanations. A video tutorial is available at: https://youtu.be/t2skgohliAc.

**Figure 5:**
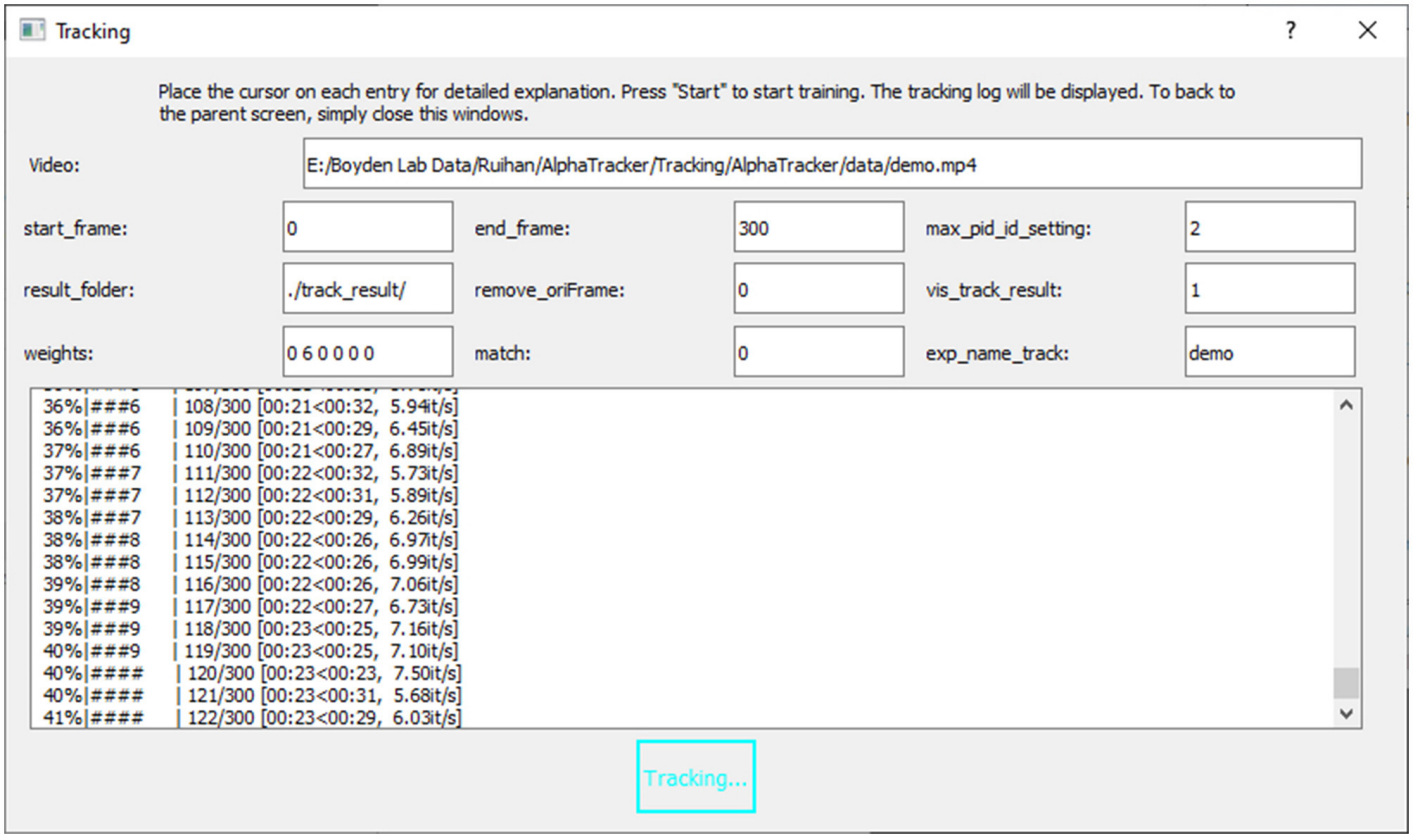
User interface for running the tracking model. The UI for running the tracking model requires an input video (“Video”) and the name of the trained model (“exp_name_track”). Users can specify the frame interval to be tracked by setting the start and end frames (“start_frame,” “end_frame”), and indicate the maximum number of mice expected in the frames (“max_pid_id_setting”). The results will be saved in the specified result folder (“result_folder”).

Important parameters are as follows:

- *video_full_path*. Path to the video
- *start_frame*. Index of the start frame of the video
- *end_frame*. Index of the last frame of the video
- *max_pid_id_setting*. Number of mice in the video
- *result_folder*. Path to the folder for saving the results
- *vis_track_result*. Whether to visualize the tracking results by overlaying the predicted keypoints on the video.
- *exp_name*. Project name. If users want to use our pretrained model, set this parameter to demo.

2. Users can start the tracking process by either clicking the “Start” button on the tracking page of the GUI (Figure 5) or by running the following in the command line.

1. conda activate alphatracker
2. python track.py

#### 2.3.5. Tracking result inspection

**Figure 6:**
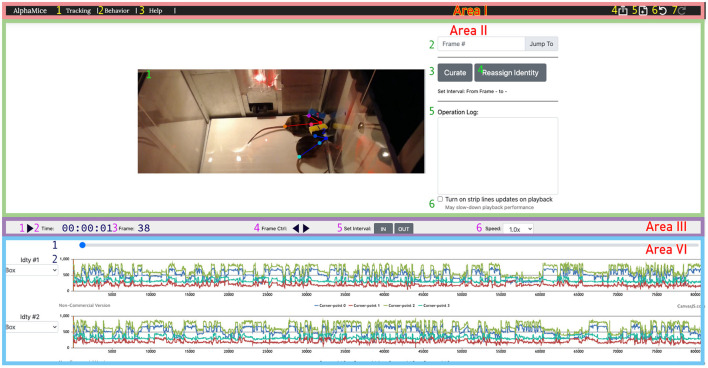
The UI for AlphaTracker allows for inspection and correction of tracking errors. The UI consists of four areas: Area I is a navigation bar with icons for navigating between different interfaces (1, 2), help function (3), undoing/redoing actions (6, 7), exporting results (4), and starting new sessions (5); The operation area (Area II) in the UI allows the user to view and edit the overlaid skeletons on the video using the cursor. It also provides the option to navigate between frames (2), initiate curation (3) and identity reassignment (3), take notes (5), and toggle the timeline's time indicator (6). Area III is a playback control panel with options for playing/pausing videos (1), displaying time and frame information (2, 3), controlling playback speed (4, 6), and specifying the interval for curation (5); Area IV is a timeline displaying the progress of the video (1), and keypoint locations over time (2). Detailed instructions for using the UI can be found on GitHub.

Users can inspect and modify the tracking results with a browser-based UI (Figure 6, Supplementary Video 6). We recommend Google Chrome as the default browser for using the UI. Pre-installed Python3 is required as Python scripts are called by the backend of the UI.

1. Users can start the UI by clicking the “Results” button on the main GUI and clicking the “Start” button on the next page. A video tutorial is available at: https://youtu.be/9Ksb04s8mm4. Alternatively, users can run the following in the command line.

1. cd UI/
2. python setup.py

2. A window should now appear in the user's browser. Click html/ and select curate.html. Click the “Click Here” button to upload the JSON tracking result (e.g., alphapose-results-forvis-tracked.json). Click the second “Click Here” button to upload the original video (e.g., demo.mp4). Specify the frame rate of the imported video. The default frame rate is 25.0 fps.

The video player allows the users to browse the videos with overlaying tracked keypoints and identities indicated by colors. Users can jump to frames of interest or scan through videos frame by frame. We provide speed control to allow flexible navigation within each video. The timeline visualizes the position of different keypoints. An abrupt change in keypoint position often suggests an error in tracking.

3. If the detected keypoints show large jitters, this indicates that the SPPE (single perspective pose estimator) model may not be properly trained. Users can return to the training stage, modify the learning rate and the number of training epochs, or provide more training data, and retrain the network.

4. Users can correct small errors such as mislabeled identities and mislabeled keypoints. To correct mislabeled keypoints, users can pause at the relevant frame(s) and drag each mislabeled keypoint to the correct position. To correct mislabeled identities, users can exchange the identities between two mice. Since errors are likely to persist after the newly corrected frame, users can select a time interval by clicking “IN” at the start of the time interval (typically, this is the frame being just modified) and “OUT” at the end of the interval. Click “curate” to update the prediction for all the frames in the interval.

5. After finishing modifying the tracking results, users can export the current clip information as a local JSON file by clicking the “export” icon.

#### 2.3.6. Behavioral clustering

**Figure 7:**
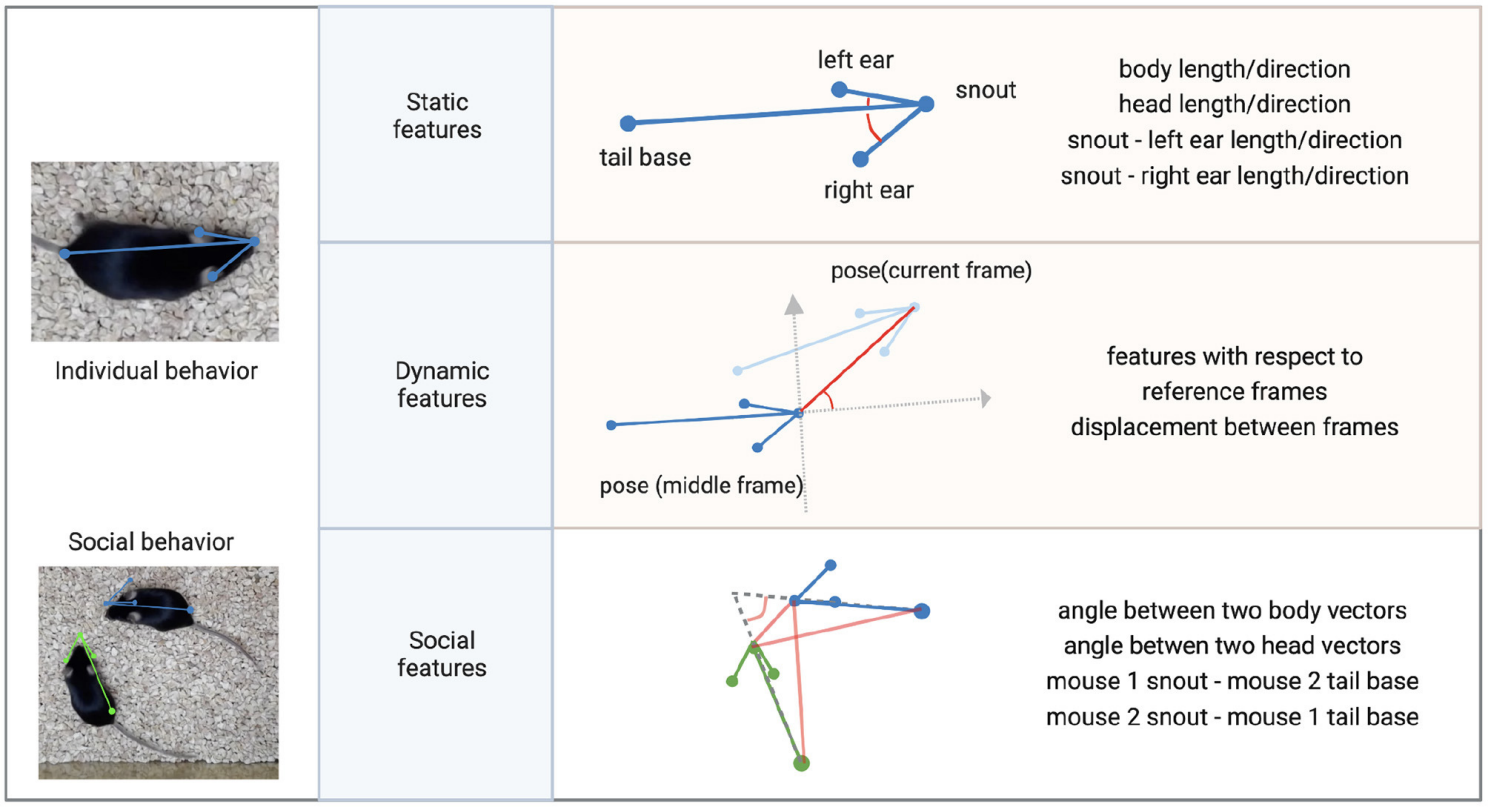
Features for clustering individual and social behavior. Individual behavioral clustering depends on both static features in individual frames and dynamic features across frames. Social behavioral clustering also depends on additional social features. The definition of example features is depicted in the diagram. Common features include distances between keypoints and the angle between two vectors. (Blue, green: skeleton of mouse poses. Red: distances between two points or angles between two vectors. Gray: reference coordinates).

AlphaTracker allows the analysis of both individual and social behavior. Here, using videos of two interacting mice, we demonstrate the ability of AlphaTracker to track animals in both scenarios. We consider clips with 15 frames (500 ms) as the unit for mouse behavior because previous research has shown that fast mouse pose dynamics can be grouped into meaningful blocks lasting 200–900 ms sub-second timescale (Wiltschko et al., 2015). For computing social features, we first rotate and move the poses such that the body of the reference mouse at the middle frame of the clip lies on the positive x axis. Figure 7 illustrates the definition of several features.

1. The success of behavioral clustering depends on the weights assigned to each feature. Users can assign higher weights to features of interest. Users either use the GUI as described in the “tracking section” or set the parameters in the ./BehavioralClustering/setting.py. Definitions of the features are listed below.

- *body_length*, body length of the reference mouse.
- *body_change_sin*, change in body direction of the reference mouse between adjacent frames.
- *left_ear*, distance between the snout and the left ear keypoints of the reference mouse.
- *left_ear_cos*, angle between the snout-left ear vector and the body vector(cos) of the reference mouse.
- *left_ear_sin*, angle between the snout-left ear vector and the body vector(sin) of the reference mouse.
- *right_ear*, distance between the snout and the right ear keypoints of the reference mouse.
- *right_ear_cos*, angle between the snout-right ear vector and the body vector(cos) of the reference mouse.
- *right_ear_sin*, angle between the snout-right ear vector and the body vector(sin) of the reference mouse.
- *displace_rho*, displacement between adjacent frames of the reference mouse.
- *displace_sin*, direction of displacement between adjacent frames(sin) of the reference mouse.
- *displace_cos*, direction of displacement between adjacent frames(cos) of the reference mouse.
- *body_length_TO*, body length of the non-reference mouse.
- *body_change_sin_TO*, change in body direction between adjacent frames of the non-reference mouse.
- *left_ear_TO*, distance between the snout and the left ear keypoints of the non-reference mouse.
- *left_ear_cos_TO*, angle between the snout-left ear vector and the body vector (cos) of the non-reference mouse.
- *left_ear_sin_TO*, angle between the snout-left ear vector and the body vector (sin) of the non-reference mouse.
- *right_ear_TO*, distance between the snout and the right ear keypoints of the non-reference mouse.
- *right_ear_cos_TO*, angle between the snout-right ear vector and the body vector(cos) of the non-reference mouse.
- *right_ear_sin_TO*, angle between the snout-right ear vector and the body vector(sin) of the non-reference mouse.
- *displace_rho_TO*, displacement between adjacent frames of the non-reference mouse.
- *displace_sin_TO*, direction of displacement between adjacent frames(sin) of the non-reference mouse.
- *displace_cos_TO*, direction of displacement between adjacent frames(cos) of the non-reference mouse.
- *two_body_sin*, angle between two body vectors(sin).
- *two_body_cos*, angle between two body vectors(cos).
- *two_head_sin*, angle between two head vectors(sin).
- *two_head_cos*, angle between two head vectors(cos).
- *TM_nose_RM_tail_rho*, distance between the tail base of the reference mouse and the snout of the non-reference mouse.
- *TM_nose_RM_tail_sin*, direction of the tail base of the reference mouse—the snout of the non-reference mouse vector(sin).
- *TM_nose_RM_tail_cos*, direction of the tail base of the reference mouse—the snout of the non-reference mouse vector (cos).
- *RM_nose_TM_tail_rho*, distance between the snout of the reference mouse and the tail base of the non-reference mouse.
- *RM_nose_TM_tail_sin*, direction of the snout of the reference mouse—the tail base of the non-reference mouse vector(sin).
- *RM_nose_TM_tail_cos*, direction of the snout of the reference mouse—the tail base of the non-reference mouse vector(cos).
- *nose_nose_rho*, distance between the two snouts.
- *nose_nose_sin*, direction of the snout-snout vector(sin).
- *nose_nose_cos*, direction of the snout-snout vector(cos).

2. (Optional) Users can define new features for clustering. We provide five intermediate variables to facilitate the computation of new features. Each variable is a NumPy array with the shape of (number_of_clip, number_of_frames_in_one_clip, number_of_key_point, 3):

- *pose_clips*, keypoints of the reference mouse.
- *pose_clips_align*, keypoints of the target mouse aligned to its middle frame.
- *poseTheOther_clips*, keypoints of the non-reference mouse.
- *poseTheOther_clips_alignSelf* , keypoints of the non-reference mouse aligned to itself in the middle frame.
- *poseTheOther_clips_alignToOther*, keypoints of the non-reference mouse aligned to the reference mouse in the middle frame.

Each new feature should be defined in ./fft_utils.py:

1. new Feature = np.ones (pose_clips.shape[1])
2. if 'newFeatureName' in feature_clips_dict:
3. feature_clips_dict['newFeatureName']. append(newFeature)
4. else:
5. feature_clips_dict['newFeatureName'] = [newFeature]

Next, the weight and normalization method of the new feature should be defined in ./utils_file/setting.py:

1. self.cluster_arg = [
2. 'thred':30,
3. 'name':'all_twoMice',
4. 'evaluation_metric':'Adjusted Rand index',
5. 'features_arg':[
6. # add the setting of the new feature here
7. 'feat_key':'newFeatureName','weight': 4,'norm':'zscore_all',
8. #......(original features).....
9. 'feat_key':'body_length','weight':1, 'norm':'zscore_all', ] ]

3. Specify the settings for behavioral clustering. If you are clustering individual behavior, change the distance threshold to be larger than the cage diameter, and set the weight for social behavior features to 0.

- *imgdir*, path to frames generated by the tracking model. Alternatively, you can set a directory of interest here and generate frames from videos by running ./BehavioralCluster/0_video2image.py.
- *tracked_json*, path to the corresponding tracking results.
- *videodir*, path to the original videos (required if you do not have the generated frames).
- *start_frame*, a list of starting frames for each video.
- *end_frame*, a list of finishing frames for each video. A number larger than the total frame number will be treated as the ending frame of the video.
- *mice_num*, number of animals in each frame.
- *joint_num*, number of keypoints per animal.
- *three*, threshold for defining clusters based on the dendrogram. Users can set this threshold to any number on their first trial, redefine this variable based on the generated dendrogram, and rerun the clustering script.
- *video_name_suffix*, suffix for the generated videos with raw videos, aligned images, feature distribution, and UMAP shown together.
- *result_folder*, the result folder for saving important intermediate results for inspection.

4. Run behavioral clustering with the following commands:

1. cd ./BehavioralClustering/
2. python fft_main_sep_twoMiceInteract.py

5. Inspect clustering results by generating the following plots with ./BehavioralClustering/Evaluation/Analysis.ipynb. Detailed instructions are included in this Jupyter Notebook. This Jupyter Notebook will generate the following plots to help determine the clustering quality with the chosen features and feature weights. Users should try different clustering thresholds and check the dendrogram and feature heatmaps in order to identify the optimal threshold to use.

- Dendrogram. The dendrogram is based on a linkage matrix calculated by the clustering algorithm. The branches of the dendrogram below the user-defined threshold are colored to indicate their cluster assignment.
- Timeline. The timeline plot displays the cluster assignments for each clip, with their color matching the cluster assignment as in the dendrogram.
- Feature heatmap. The feature heatmap visualizes the relative strength of each feature in the cluster.
- UMAP. The UMAP shows the topological structure of all the clips in the feature space. Each dot represents one cluster, colored by their cluster assignment.
- Mutual information plots. The mutual information plot quantifies the mutual information between each feature and the cluster assignment. Note: Larger mutual information suggests the feature is a strong marker of the cluster.
- Similarity matrix between clusters. Note: Clusters with a high similarity score are hard to differentiate.
- Representative skeleton for each skeleton. Cluster skeletons visualize the representative pose and its temporal evolution for each cluster.

6. Besides these analysis plots, users can also inspect generated videos saved at *self.gen_video_folder* as specified in setting.py with feature and cluster assignment.

7. Once the optimal threshold is identified, users should set the threshold in the setting.py and rerun the algorithm. This will save the correct cluster information for the clustering UI.

#### 2.3.7. Clustering UI

**Figure 8:**
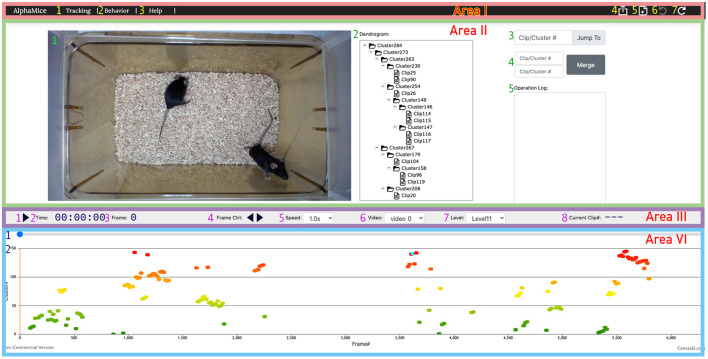
The UI for AlphaTracker provides the ability to inspect and correct clustering errors. Area I is a navigation bar with options for navigation (1, 2), help (3), undoing/redoing actions (6, 7), result export (4), and starting new sessions (5). Area II displays the video (1) and the dendrogram (2) of clustering results, along with frame navigation (3). Double-clicking a node in the dendrogram highlights all corresponding frames in the timeline in cyan. Double-clicking a node in the dendrogramwould highlight all the frames belonging to that cluster in the timeline. Right-clicking allows for cluster and clip manipulation (move, rename, delete). Merging of clusters is available in region 4, with the ability to record rationale in region 5. Area III contains a playback control panel with options to play/pause video (1), display time, frame, and clip information (2, 3, 8), control playback speed (4, 5), choose video (6), and set the level to expand/collapse in the dendrogram (7). Area IV displays the progress bar (1) and cluster assignments for each clip encoded by color (2). Detailed instructions for using the UI can be found on GitHub.

We provide a Clustering UI for inspecting the clustering results (Figure 8).

1. Open the clustering UI following the same instruction as the tracking UI. Choose cluster.html. Upload the JSON file that contains the clip information (clips_info.json) generated in the behavioral clustering step. Import the original video and specify the frame rate. Upload the JSON file that contains the cluster structure (e.g., Z_all_twoMice.json).
2. Play the video and inspect the cluster assignment for each clip. Users can examine the dendrogram by expanding and collapsing the tree structure. The branches of the dendrogram can be merged and moved to modify the cluster assignment. A detailed explanation is provided in our GitHub manual.

#### 2.3.8. Tracking with Google Colab

In addition to the desktop version, we also provide a Colab notebook for training and tracking. Users looking for a quick test of AlphaTracker can open this notebook https://colab.research.google.com/drive/1wYBAj3kjLMe6uir3TJVfWRAJNHtjCaPG and simply run through all blocks following the instructions. If users would like to train their own model, we provide another notebook https://colab.research.google.com/drive/1bGUo3eMWIfzXiFWCvNrNiTOzhSsTsVHV.

1. Open the Colab notebook and save a copy to your personal Google Drive.
2. Click Runtime and then change the runtime type to “GPU.” Run the “Install” section to connect to your Google Drive. Your Google Drive will now be mounted at /content/drive/MyDrive. Run the next block to download AlphaTracker and finish installation.
3. Upload your annotated training datasets to Google Drive and set variables such as “image_root_list,” “json_file_path,” “number_of_animals,” “number_of_poses,” “video_path” in the “Setting” section.
4. Run the training code block if you would like to train the model with your own datasets.
5. Run the tracking code block to perform training on the videos you listed in setting.py or the default demo video. Once this step is complete, in order to inspect the tracking results, you can go to the result folder as specified in setting.py.

## 3. Results

### 3.1. Anticipated tracking results

To quantify AlphaTracker's performance and compare it to SLEAP and DeepLabCut, we conducted experiments using a mouse dataset where. Trained human annotators labeled the bounding box and four keypoints (snout, left ear, right ear, and tail base) for each mouse in each frame. Our customization of the DeepLabCut default model includes the following modifications: enabling automatic computation of the PAF graph, utilization of the box tracker, and setting the maximum number of iterations to 100,000. Our customization of the SLEAP default model includes several modifications to improve its tracking performance. Firstly, we used the bottom-up model and set the tracker mode as “flow”. Secondly, we implemented culling with an IoU threshold of 0.8. Thirdly, we utilized the instance similarity method and the greedy matching method. Fourthly, we set the elapsed window to 5 and used a robust quantile of similarity scores of 0.95. Fifthly, we applied post-tracking break connection to improve tracking continuity. Finally, we adjusted the minimum and maximum rotation angles to -180 and 180 degrees, respectively. We used the standard CLEAR MOT metrics [Average Precision (AP), Multiple Object Tracking Accuracy (MOTA), and Multiple Object Tracking Precision (MOTP)] (Bernardin and Stiefelhagen, 2008), and evaluated the performance using the open-source Poseval tool (Pishchulin et al., 2015) AP assesses the accuracy of object detection by computing precision and recall values. MOTA evaluates three types of errors: missed objects in a sequence, false positives, and mismatches. MOTP calculates the average total position error for matched object-hypothesis pairs across all frames. The evaluation was performed using the open-source Poseval tool (Pishchulin et al., 2015). To adapt the MOT metrics for mouse tracking, we modified the threshold for distinguishing matched keypoints from mismatched keypoints to be 5% of the bounding box's diagonal.

**Figure 9:**
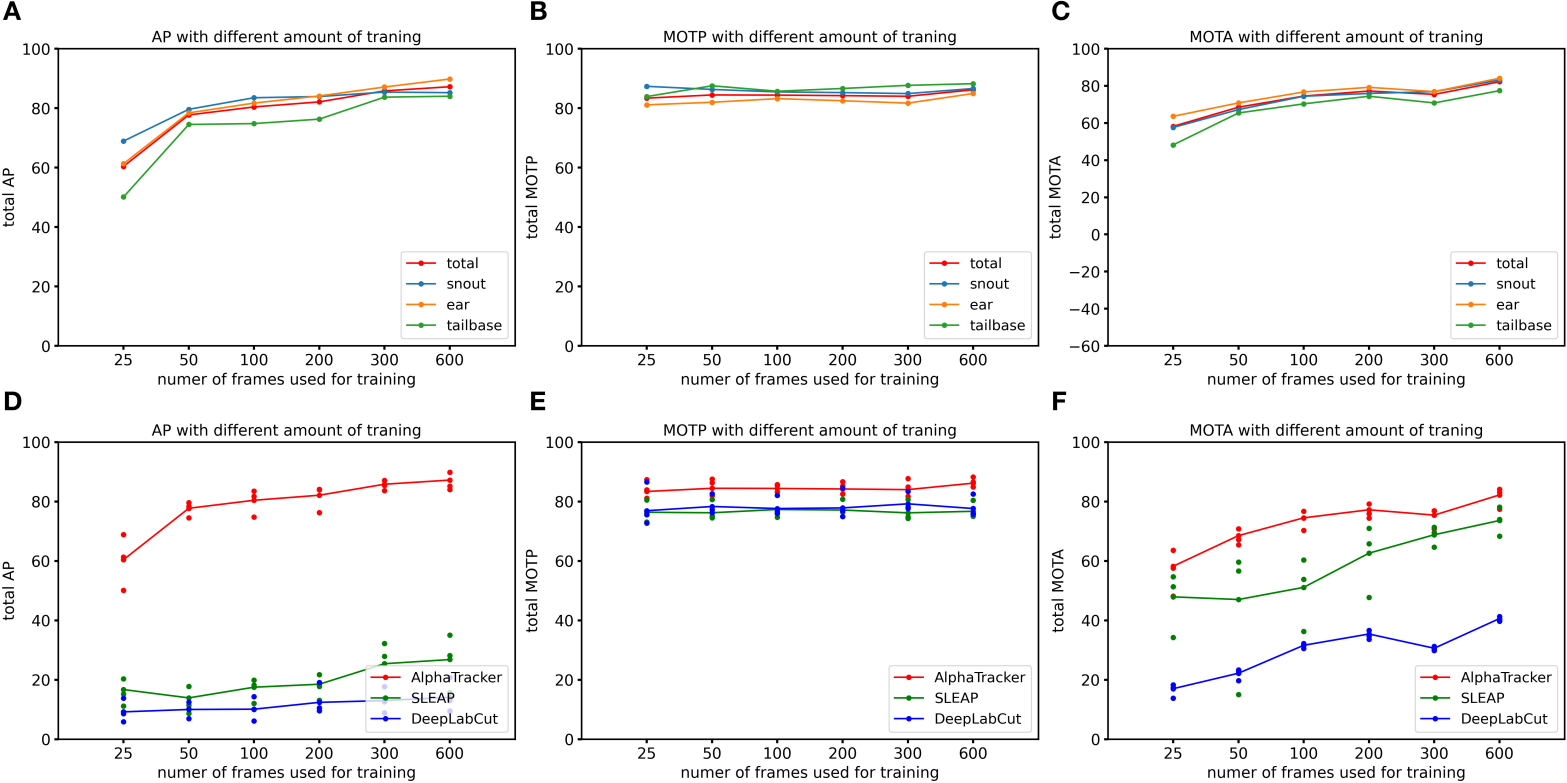
AlphaTracker surpasses the performance of both DeepLabCut and SLEAP in tracking two mice in a home cage. **(A–C)** Show the Average Precision (AP), Multi-Object Tracking Precision (MOTP), and Multi-Object Tracking Accuracy (MOTA) metrics for different keypoints of AlphaTracker results. The metrics were evaluated for different amounts of training frames (25, 50, 100, 200, 300, and 600 frames) using a 200-frame evaluation dataset. Different colors represent different keypoints including snouts (blue), ears (yellow) and tailbases (green) and the total metrics (red). **(D–F)** Show the evaluation results for AlphaTracker (red), SLEAP (green), and DeepLabCut (blue), with connected dots representing the total metric and the unconnected dots representing the metric for individual body parts.

We evaluated AlphaTracker's performance on a dataset with two mice interacting in a home cage, recorded at a resolution of 1,920 × 1,080 p. Results shown in Table 1 indicate that AlphaTracker outperformed SLEAP and DeepLabCut in terms of mAP, MOTA, and MOTP when trained with 600 frames and tested on 200 held-out frames (Supplementary Video 1). Furthermore, AlphaTracker demonstrate consistent performance across all four keypoints, while SLEAP and DeepLabCut showed significant variance, as shown in Figure 9. Furthermore, AlphaTracker showed high performance with only 50 frames of training data, achieving an mAP higher than 0.7 (Figure 9).

**Table 1:** AlphaTracker demonstrated superior performance in tracking both two mice in a home cage and four mice in an operant chamber.

	**Two mice home cage**			**Four mice operant chamber**		
	**MOTA**	**MOTP**	**mAP**	**MOTA**	**MOTP**	**mAP**
AlphaTracker	82.2	86.2	87.2	84.0	87.2	85.6
DeepLabCut	40.6	77.6	14.1	71.4	86.5	68.0
SLEAP	73.6	76.7	26.8	77.9	86.5	83.9

AlphaTracker, DeepLabCut, and SLEAP were evaluated on two datasets: two mice in a home cage and four mice in an operant chamber. Each model was trained on 600 annotated frames and evaluated on 200 frames with human-labeled ground truth. The evaluation results show that AlphaTracker outperforms DeepLabCut and SLEAP in both datasets, achieving higher keypoint detection accuracy (mAP) and tracking consistency (MOTA and MOTP).

Moreover, we evaluated AlphaTracker's performance in tracking multiple identical-looking animals using four C57/BL6 mice in a metal operant chamber scenario. Our evaluation (Supplementary Video 2) showed that AlphaTracker outperformed SLEAP and DeepLabCut in all metrics (Table 1, Supplementary Video 2). We also tested AlphaTracker on mice with head implants, a common scenario in neuroscience research, and demonstrated its robust performance (Supplementary Video 3). This highlights AlphaTracker's potential for studying naturalistic social group dynamics in common neuroscience settings.

It's worth mentioning that the four mice operant chamber dataset was collected with low-quality webcams at a resolution of 540 p. AlphaTracker demonstrated robust performance in tracking animals in these videos (Table 1), making it an attractive solution for large-scale animal behavior studies as it enables the monitoring of multiple cages using low-cost webcams, greatly reducing the overall cost.

### 3.2. Anticipated behavioral clustering results

**Figure 10:**
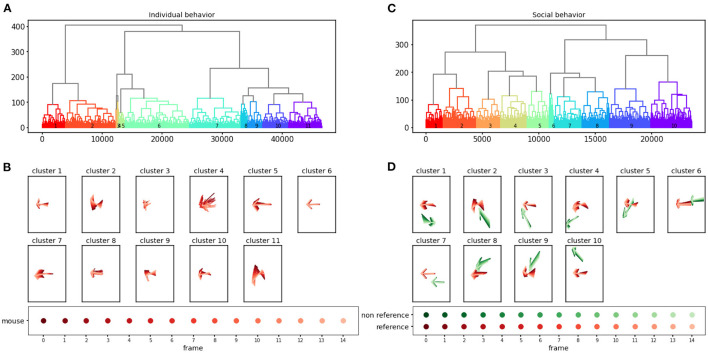
AlphaTracker identifies clusters for both individual and social behavior. Hierarchical clustering was performed on 15-frame clips (500 ms duration) generated from videos of interacting dyads. The dendrogram of the clustering results is shown in **(A)** for individual behavior and **(C)** for social behavior. Each leaf on the dendrogram represents a single clip, and their relative distance reflects their similarity in the feature space. The different colors and numbers indicate the assigned cluster for each individual clip. Example skeletons in **(B, D)** provide a visual representation of the typical movement in each cluster. The pose of the reference mouse is displayed in red, while the non-reference mouse's pose is displayed in green. The movement in a 15-frame clip is illustrated by plotting a skeleton representation, with colors ranging from dark to light to denote each individual frame. The skeletons have been rotated to align the pose of the reference mouse in the central frame with the negative x-axis direction.

The behavioral clustering component of AlphaTracker enables the clustering of both individual behavior and social interaction in an unsupervised manner. Here, we analyze a total of 4,661 clips for individual behavioral clustering and 2,356 clips for social behavioral clustering collected from four videos (Figure 10, Supplementary Videos 4, 5). Our algorithm can capture the following individual behaviors: walking, digging, sniffing, rearing, turning, face grooming, and body grooming, and social behaviors: following, chasing, anogenital sniffing, face sniffing, and social rearing.

**Figure 11:**
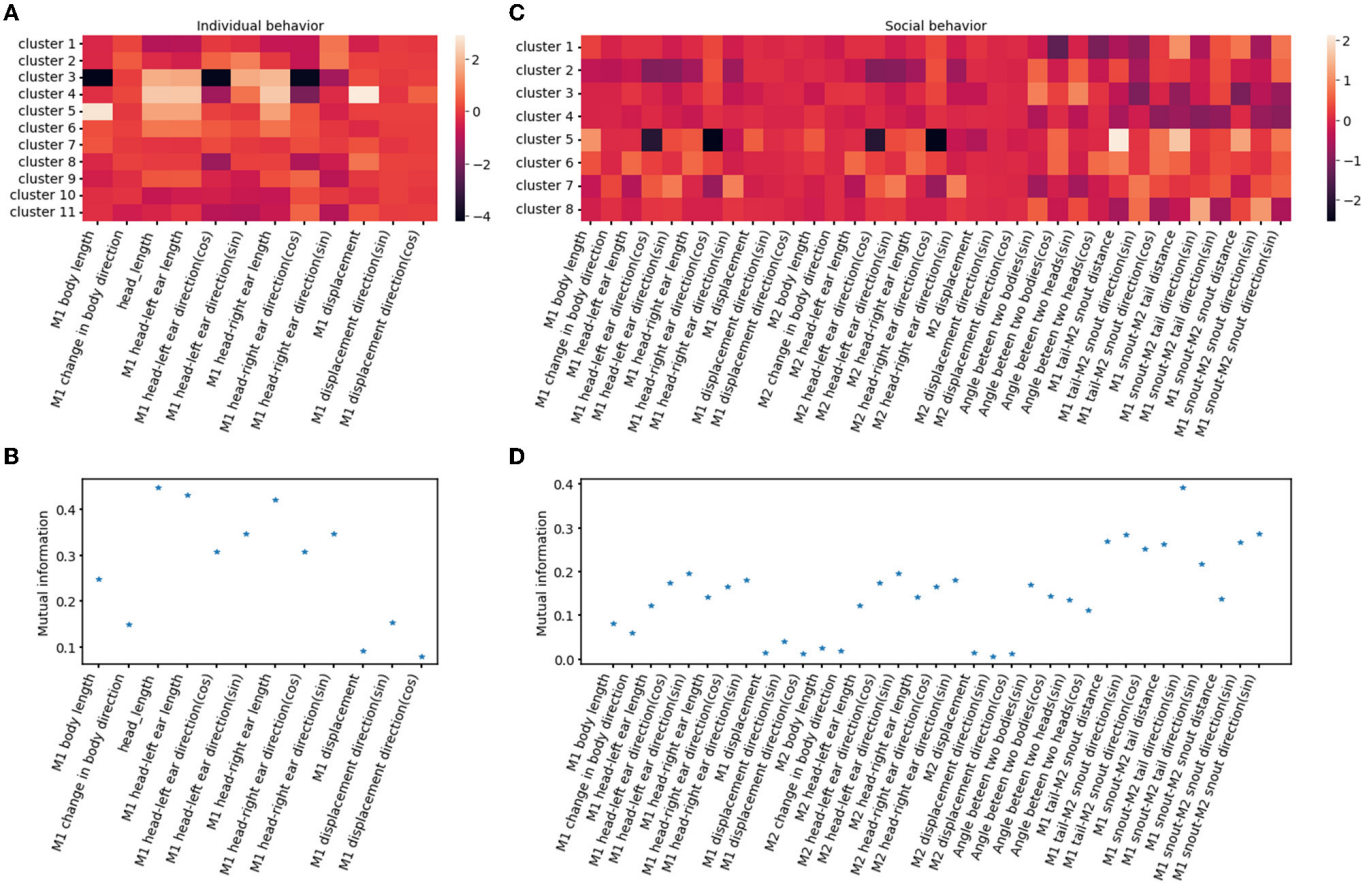
Behavioral clusters can be differentiated by unique combinations of features. **(A, C)** Illustrate the heatmaps of averaged feature values utilized for individual and social behavioral clusters, respectively. M1 refers to the reference mouse and M2 refers to the non-reference mouse in the dyad. **(B, D)** Show the mutual information between cluster assignment and each feature for individual and social behavioral clusters, respectively. A higher mutual information score indicates that the feature plays a crucial role in distinguishing specific clusters.

To evaluate the importance of each feature in clustering, we calculate the mutual information between features and cluster assignment (Figure 11), with the expectation that higher mutual information indicates that the feature may represent a unique characterization of a given cluster. For example, distances between two mice are a strong indicator of social clusters, while related to the head such as head length and nose-left ear distance stand out among other individual features, indicating the salience of the head in of many behaviors like rearing, digging and turning (Figure 11). The identified behavioral clusters allow users to visualize the temporal dynamics of animal behavior. This opens up the opportunity for associative analysis between changes in behavior motifs with experimental factors like optogenetic stimulus, drug administration, environmental changes, and manipulation in a social hierarchy.

To validate the performance of our behavioral clustering algorithm, we compared its output to the ground 488 truth of human annotation. A human scorer was trained to categorically annotate behaviors. We used the Adjusted Rand Index (ARI) to measure the similarity of the class assignment between the algorithm and human scorer. ARI scores range from -1 to 1, with negative values indicating independent labels, positive values indicating close agreement with ground truth labels, and values close to zero indicating random label assignments.

We evaluated the algorithm's performance on datasets of different sizes. The small dataset consisted of five videos, including two videos with human-annotated ground truth, with a total of 1345 clips. The large dataset included two additional videos, with a total of 3034 clips. The results presented in Table 2 suggest that both datasets perform significantly better as compared to randomly assigning clips to each cluster. Moreover, the performance of the model further improved when given a larger clustering dataset, likely due to better coverage of the continuous input space.

**Table 2:** The results of AlphaTracker's clustering algorithm are consistent with human judgement.

	**Large dataset (3,034 clips)**	**Small dataset (1,345 clips)**	**Random assignment**
Adjusted rand index	0.201186	0.186533	0.003451

The accuracy of AlphaTracker's clustering algorithm was evaluated by comparing its output with human annotations for individual clips (500 ms). The annotations included individual behaviors: walking, digging, sniffing, rearing, turning, face grooming, and body grooming, and nine social behaviors: following, chasing, anogenital sniffing, face sniffing, and social rearing. The similarity of the class assignments was measured using the Adjusted Rand Index (ARI). AlphaTracker showed significantly higher ARI compared to random assignment, demonstrating its consistency with human judgement. Additionally, using a relatively small dataset of 1,345 clips, AlphaTracker was able to accurately capture most of the cluster assignments, further highlighting its efficiency and effectiveness.

### 3.3. Timing

Installation time for AlphaTracker is highly dependent on the installation method selected and users' Internet speed. We estimate that it will take a user between 10–30 minutes to download and install the package, pre-trained model, demo data, and all dependencies on Linux. On Windows, installation may take about 2 minutes less since Windows does not support YOLO training when using the C-based darknet toolbox.

The training time for AlphaTracker (including YOLO and pose estimation) is highly dependent on hardware performance, dataset, and hyper-parameter settings. Using our default settings and example data (about 6000 images), it takes approximately 2 hours to train the YOLO detector and the pose estimation model.

The tracking time for AlphaTracker (including detection, pose estimation, and tracking) is also highly dependent on hardware performance, dataset, and hyperparameter settings. Using our default settings and demo video (about 7 minutes), tracking takes approximately 2 hours.

The time required for behavior clustering varies according to the features selected. Using keypoint-based features takes approximately 10 minutes. When using the UI to inspect the results, the main time cost is spent on loading data, which typically takes about 1–2 minutes.

These time estimates are for a server with 72 Intel(R) Xeon(R) Gold 6150 CPU @ 2.70GHz, and 393 GB of RAM, running an Ubuntu 18.04.5 LTS system, using a 2080-Ti GPU. CPU times or Windows times are also noted where appropriate.

## 4. Discussion

In this paper, we introduce AlphaTracker, a robust machine-learning pipeline that accurately tracks and estimates the poses of multiple unmarked animals. AlphaTracker also includes a feature for discovering behavioral patterns through unsupervised clustering and a user-friendly interface for visualizing and proofreading results. Our pipeline is available on GitHub for educational use and is user-friendly for non-programmers. Users can model and analyze animal behaviors in a matter of hours with a GPU. Our aim is to provide the research community with a powerful tool for high-throughput behavioral analysis.

Traditional multi-animal tracking approaches require heuristics to resolve animal identities, such as artificial colored markers (EthoVision, Noldus) and bleach-marking with fur patterns (Ohayon et al., 2013). These methods require performing procedures on animals that could affect their natural behavior. Animal tracking has benefited greatly from advances in pose estimation, such as DeepLabCut, a software package that can reliably track human-defined unique keypoints (Mathis and Mathis, 2020). A recent algorithm, Moseq, has made progress on automated behavioral identification by using a depth camera and unsupervised learning theory (Wiltschko et al., 2015). And, SimBA presents an open-source package with a graphical interface and workflow that uses pose-estimation to create supervised machine learning (Nilsson et al., 2020). However, these tools have not been effective in tracking multiple identical animals. In recent years, other tools for multiple animal tracking have emerged. As an example, SLEAP is a full-featured general-purpose multi-animal pose tracking framework tested on a diverse array of datasets representative of common social behavioral monitoring setups and designed for flexibility (Pereira et al., 2020b). Our model outperforms these tools in keypoint detection accuracy and multi-animal identification consistency which is critical for studying social behavior.

Our AlphaTracker model has two main limitations. Firstly, it was designed for tracking mice from a top view, and its adaptation to other animals and environments requires expert tuning and adaptation. To make this process easier, we have created a tutorial on annotating new data and model training. Typically, 200 annotated frames yield satisfactory performance in new settings. The second limitation is the hardware requirement for a GPU for model training. To overcome this, we offer a Google Colab version of AlphaTracker. However, the free Colab version may time out during long training sessions, and requires packages to be reinstalled and connection to Google Drive for file storage each time it is used.

Users may also encounter challenges that are common to all models of this kind. Firstly, keypoint detection accuracy may be affected by occlusions or animals temporarily leaving the frame. To address this, we have provided a curation UI for users to correct misidentification and mislabeling. Secondly, the clustering algorithm does not work well for heterogeneous videos, such as those with different imaging angles, animal sizes and scaling factors. In these cases, the algorithm will produce clusters specific to each condition, rather than uniform behavior patterns.

We envision AlphaTracker greatly facilitating systems neuroscience research, as it premiered in Padilla-Coreano et al. (2022). In that paper, AlphaTracker played a key role in furthering research studying the role of the medial prefrontal cortex in regulating social hierarchy. Besides this paper, there has been a recent increase in the study of the neural mechanisms behind behaviors such as social dominance, mating behavior, and maternal behavior. To fully understand these behaviors, it is important to have reliable and efficient methods of tracking social interactions and quantifying behavioral patterns with minimal bias. Human annotation performed by multiple researchers suffers from biases in subjective behavior annotation and intensive labor. AlphaTracker which is designed for reducing biases and elevating efficiency holds great potential in accelerating this field.

## Data availability statement

The original contributions presented in the study are included in the article/Supplementary material, further inquiries can be directed to the corresponding authors.

## Ethics statement

The animal study was reviewed and approved by IACUC Salk Institute and MIT.

## Author contributions

ZC, RZ, and H-SF contributed to the conceptualization. ZC, RZ, HZhu, HZho, and YZ wrote the code. RZ and NP-C collected the mouse data. RR and H-SF labeled groundtruth data. RZ, H-SF, ZC, HZhu, YZ, NP-C, and HZho wrote the manuscript with input from all authors. CL and KT supervised the project. All authors contributed to the article and approved the submitted version.

## References

[B1] Berman G. J. (2018). Measuring behavior across scales. BMC Biol. 16, 1–11. 10.1186/s12915-018-0494-7 29475451 PMC5824583

[B2] Bernardin K. Stiefelhagen R. (2008). Evaluating multiple object tracking performance: the CLEAR MOT metrics. EURASIP J. Image Video Process. 2008:246309. 10.1155/2008/24630928273796

[B3] Chen L. Ai H. Zhunag Z. Shang C. (2018). “Real-time multiple people tracking with deeply learned candidate selection and person re-identification,” in 2018 IEEE International Conference on Multimedia and Expo (ICME) (IEEE), 1–6. Available online at: https://scholar.google.com/scholar?hl=en&as_sdt=0%2C22&q=Real-time+multiple+people+tracking+with+deeply+learned+candidate+selection+and+person+Re-Identification&btnG=

[B4] Darwin C. (1872). The Expression of the Emotions in Man and Animals. London: John Murray. 10.1037/10001-000

[B5] Fang H.-S. Li J. Tang H. Xu C. Zhu H. Xiu Y. . (2022). Alphapose: Whole-body regional multi-person pose estimation and tracking in real-time. IEEE Trans. Pattern Anal. Mach. Intell. 10.1109/TPAMI.2022.3222784 37145952

[B6] Fang H.-S. Xie S. Tai Y.-W. Lu C. (2017). “RMPE: regional multi-person pose estimation,” in Proceedings of the IEEE International Conference on Computer Vision, 2334–2343. 10.1109/ICCV.2017.256

[B7] Feng W. Hu Z. Wu W. Yan J. Ouyang W. (2019). “Multi-object tracking with multiple cues and switcher-aware classification,” in 2022 International Conference on Digital Image Computing: Techniques and Applications (DICTA). p. 1–10. Available online at: https://www.semanticscholar.org/paper/Multi-Object-Tracking-with-Multiple-Cues-and-Feng-Hu/2daffd29687138888f4bfdd4f597eb8a21cac57d

[B8] Hu J. Li S. Gang S. Albanie S. (2017). Squeeze-and-excitation networks. IEEE Trans. Pattern Anal. Mach. Intell. 42, 2011–2023. 10.1109/TPAMI.2019.2913372 31034408

[B9] Kabra M. Robie A. A. Rivera-Alba M. Branson S. Branson K. (2013). JAABA: interactive machine learning for automatic annotation of animal behavior. Nat. Methods 10, 64–67. 10.1038/nmeth.2281 23202433

[B10] Kalman R. E. (1960). A new approach to linear filtering and prediction problems. J. Basic Eng. 82, 35–45. 10.1115/1.3662552

[B11] Mathis A. Mamidanna P. Cury K. M. Abe T. Murthy V. N. Mathis M. W. . (2018). DeepLabCut: markerless pose estimation of user-defined body parts with deep learning. Nat. Neurosci. 21, 1281–1289. 10.1038/s41593-018-0209-y 30127430

[B12] Mathis M. W. Mathis A. (2020). Deep learning tools for the measurement of animal behavior in neuroscience. Curr. Opin. Neurobiol. 60, 1. 10.1016/j.conb.2019.10.008 31791006

[B13] Nilsson S. R. O. Goodwin N. L. Choong J. J. Hwang S. Wright H. R. Norville Z. C. . (2020). Simple behavioral analysis (SimBA)-an open source toolkit for computer classification of complex social behaviors in experimental animals. BioRxiv. 10.1101/2020.04.19.049452

[B14] Ohayon S. Avni O. Taylor A. L. Perona P. Roian Egnor S. E. (2013). Automated multi-day tracking of marked mice for the analysis of social behaviour. J. Neurosci. Methods 219, 10–19. 10.1016/j.jneumeth.2013.05.013 23810825 PMC3762481

[B15] Padilla-Coreano N. Batra K. Patarino M. Chen Z. Rock R. R. Zhang R. . (2022). Cortical ensembles orchestrate social competition through hypothalamic outputs. Nature 603, 667–671. 10.1038/s41586-022-04507-5 35296862 PMC9576144

[B16] Pereira T. D. Shaevitz J. W. Murthy M. (2020a). Quantifying behavior to understand the brain. Nat. Neurosci. 23, 1537–1549. 10.1038/s41593-020-00734-z 33169033 PMC7780298

[B17] Pereira T. D. Tabris N. Li J. Ravindranath S. Papadoyannis E. S. Wang Z. Y. . (2020b). SLEAP: multi-animal pose tracking. BioRxiv. 10.1101/2020.08.31.276246

[B18] Pishchulin L. Insafutdinov E. Tang S. Andres B. Andriluka M. Gehler P. V. . (2015). “Deepcut: Joint subset partition and labeling for multi person pose estimation,” in Proceedings of the IEEE Conference on Computer Vision and Pattern Recognition (IEEE), 4929–4937. Available online at: https://www.cv-foundation.org/openaccess/content_cvpr_2016/html/Pishchulin_DeepCut_Joint_Subset_CVPR_2016_paper.html

[B19] Redmon J. Farhadi A. (2018). Yolov3: An incremental improvement. arXiv [Preprint]. arXiv: 1804.02767. Available online at: https://arxiv.org/abs/1804.02767

[B20] Ristani E. Tomasi C. (2018). Features for multi-target multi-camera tracking and re-identification. 10.1109/CVPR.2018.00632

[B21] Tinbergen N. (1963). On aims and methods of ethology. Zeitschr. Tierpsychol. 20, 410–433. 10.1111/j.1439-0310.1963.tb01161.x

[B22] Ward J. H. (1963). Hierarchical grouping to optimize an objective function. J. Am. Stat. Assoc. 58, 236–244. 10.1080/01621459.1963.10500845

[B23] Wiltschko A. B. Johnson M. J. Iurilli G. Peterson R. E. Katon J. M. Pashkovski S. L. . (2015). Mapping sub-second structure in mouse behavior. Neuron 88, 1121–1135. 10.1016/j.neuron.2015.11.031 26687221 PMC4708087

[B24] Wiltschko A. B. Tsukahara T. Zeine A. Anyoha R. Gillis W. F. Markowitz J. E. . (2020). Revealing the structure of pharmacobehavioral space through motion sequencing. Nat. Neurosci. 23, 1433. 10.1038/s41593-020-00706-3 32958923 PMC7606807

