# Supplementary material for: AlphaTracker: a multi-animal tracking and behavioral analysis tool
Source: Front Behav Neurosci. 2023 May 30;17:1111908. doi: 10.3389/fnbeh.2023.1111908 (PMC10266280; doi:10.3389/fnbeh.2023.1111908)
Supplement: Supplementary file 7 [file Data_Sheet_1.PDF]

Supplementary Material

1 SUPPLEMENTARY VIDEOS

- **Supplementary video 1** A video tutorial for using the tracking UI.
- **Supplementary video 2** A video showing tracking performance of AlphaTracker for two mice in home cages, taken at 1080p resolution. The mice's skeletons are defined by connections between the snout and left ear, the snout and right ear, and the snout and tail base, with a number indicating each mouse's identity. The video was evaluated on 200 human-labeled ground truth frames, and the resulting metrics are mAP: 98.387.2, MOTA: 82.2, and MOTP: 86.2.
- **Supplementary video 3** A video showing the tracking performance of AlphaTracker for four mice in a metal operant chamber, taken at 540p. The mice's skeletons are defined by connections between the snout and left ear, the snout and right ear, and the snout and tail base, with a number indicating each mouse's identity. The video was evaluated on 200 human-labeled ground truth frames, and the resulting metrics are mAP: 85.6, MOTA: 84.0 MOTP: 87.2.
- **Supplementary video 4** A video showing the tracking performance of AlphaTracker for two mice with headstage in the operant chamber. The video was taken with a low resolution webcam (576p) . The skeleton for each mouse consists of the connection between snout and left ear, snout and right ear, and snout and tail base. The identity of each mouse is indicated by a number next to its snout.
- **Supplementary video 5** A video showing behavioral clustering results for individual behavior. We include both the original video and the cropped videos with one mouse as the reference frame (either the reference mouse or the target mouse). The dendrogram and the UMAP show the global cluster assignment with the clips in the same cluster represented by the same color. The red line in the dendrogram and the red star in the UMAP indicate the current clip for display. The bar plot shows the normalized value for all features. Note: we include all the features here, not just the features specified in `setting.py`.
- **Supplementary video 6** A video showing behavioral clustering results for social behavior between two mice. Both the original video and the cropped regions centered on the reference mouse in the dyad are included. The dendrogram and UMAP visualization highlight the global cluster assignments with clips in the same cluster represented by the same color. The red line in the dendrogram and red star in the UMAP highlight the current clip being displayed. A bar plot displays the normalized values for all features.
